# A pedometer based physical activity self-management program for children and adolescents with physical disability – design and methods of the StepUp study

**DOI:** 10.1186/1471-2431-14-31

**Published:** 2014-02-03

**Authors:** Carol Maher, Angela Crettenden, Kerry Evans, Myra Thiessen, Monica Toohey, Jim Dollman

**Affiliations:** 1 Health and Use of Time Group, School of Health Sciences, University of South Australia, GPO Box 2471, Adelaide, South Australia 5001, Australia; 2 Novita Children’s Services, 171 Days Road, Regency Park, South Australia 5010, Australia; 3 School of Art, Architecture and Design, University of South Australia, GPO Box 2471, Adelaide, South Australia 5001, Australia; 4 School of Health Sciences, University of South Australia, GPO Box 2471, Adelaide, South Australia 5001, Australia

**Keywords:** Physical activity, Disability, Intervention

## Abstract

**Background:**

Physical activity affords a wide range of physiological and psychological benefits for children and adolescents, yet many children with physical disabilities are insufficiently active to achieve these benefits. The StepUp program is a newly developed 6-week pedometer-based self-management program for children and adolescents with physical disability. Participants use a pedometer to undertake a 6-week physical activity challenge, with personalised daily step count goals set in consultation with a physiotherapist. The study aims to evaluate the effectiveness of the StepUp program, using a randomised control trial design.

**Methods/design:**

A target sample of 70 young people with physical disabilities (aged 8–17 years, ambulant with or without aid, residing in Adelaide) will be recruited. Participants will be randomly allocated to either intervention or control following completion of baseline assessments. Assessments are repeated at 8 weeks (immediately post intervention) and 20 weeks (12 weeks post intervention). The primary outcome is objective physical activity determined from 7 day accelerometry, and the secondary outcomes are exercise intention, physical self-worth, quality of life and fatigue. Analyses will be undertaken on an intention-to-treat basis using random effects mixed modelling.

**Discussion:**

This study will provide information about the potential of a low-touch and low-cost physical activity intervention for children and adolescents with cerebral palsy.

**Trial registration:**

Australian New Zealand Clinical Trials Registry (ANZCTR): ACTRN12613000023752.

## Background

Physical activity affords wide-ranging physiological and psychological benefits for children and adolescents, regardless of disability status [1]. However, regular participation in physical activity appears to hold important additional benefits for individuals with physical disabilities. For example, evidence is emerging that physical activity is vital for prevention of deterioration in physical function and independence in young and middle aged adults with cerebral palsy [2-4]. Furthermore, physical activity plays a key role in the management of chronic health conditions such as asthma [5] and heart disease [6], and these are common comorbidities in children with physical disabilities [7].

Up to 3.7% of Australian children have a physical disability [7], in many cases affecting their ability to participate in everyday life. Therapy and health services for young people with disabilities have traditionally focussed on secondary prevention. A focus on physical activity, per se, for young people with physical disabilities is a relatively fledgling field of research. Recent research has shown that young people with cerebral palsy and other physical disabilities are less physically active than their non-disabled peers [8,9]. It is also clear that young people with disabilities tend to participate in lower intensity physical activities compared with children without disabilities [10].

To date, just a handful of physical activity interventions for young people with disabilities have been reported in the scientific literature [11-14]. Ideally, such programs need to be flexible, in order to accommodate the wide range of participants’ gross motor abilities and interests. Additionally, young people with physical disabilities are often geographically dispersed and face transport barriers, therefore programs which can be undertaken without intensive face-to-face contact with therapists or fitness instructors are advantageous. Furthermore, programs requiring minimal equipment and staffing resources offer considerable potential to be incorporated into ongoing services provided by therapists and health providers.

Pedometers are small devices, typically worn on the waist band which measure step counts [15]. Pedometer-based interventions are a simple and effective means of increasing physical activity, with a recent systematic review of pedometer-based interventions in youth without disabilities finding that 12 out of 14 studies produced significant increases in physical activity [16]. Furthermore, development of self-management skills is believed to enhance well-being, self-determination and participation in health care, ultimately leading to improved health outcomes, as well as reduced health care utilisation (and associated costs) [17]. To address these issues, we have developed a six-week self-management, pedometer-based physical activity program for young people with physical disabilities, titled “StepUp”.

This study aims to evaluate this new six-week pedometer-based self-management program for ambulant children and adolescents with physical disabilities. Specifically, it aims to determine (a) whether the program is effective in increasing physical activity over the course of the 6 week intervention and at longer-term follow up, (b) whether the program impacts physical self-worth, exercise intention, pain and fatigue and quality of life, and (c) the program’s acceptability and engagement.

## Methods/design

### Study design

The StepUp study is a two group (StepUp intervention versus standard care) randomised controlled trial with data collection at three time points; at baseline, end of intervention (8 weeks post baseline) and follow up (20 weeks post baseline; 12 weeks post end of intervention). Ethical approval for the study has been granted by the University of South Australia Human Research Ethics Committee and the study protocol has been registered with the Australian and New Zealand clinical trials registry and assigned the protocol number: ACTRN12613000023752.

### Study sample

The study will aim to enrol 50–70 participants. Participants are being recruited through Novita Children’s Services, the major provider of community-based therapy, equipment and family support services to young people with disabilities and special needs aged 0–18 years in the state of South Australia. Novita clients are eligible to participate if they (1) have a physical disability, (2) are aged 8–17 years, (3) have mild-to-moderate levels of physical disability (able to ambulate in the community with or without assistance; Gillette Functional Assessment Questionnaire levels 7, 8, 9 or 10 [18]), (4) live in or near Adelaide, Australia, and (5) are considered by their parent to have cognitive ability to understand the program. Exclusion criteria are (1) recent or planned medical and/or orthopaedic intervention (e.g. surgery or botulinim toxin injections) impacting ability to be physically active, and (2) injury impacting on ability to partake in physical activity. Recruitment is taking place on a rolling basis from June to December 2013.

### Procedure

Potential participants identified from the Novita client database are being sent an informative invitation letter. Participants and their parents are required to give written informed consent to be involved in the study.

### Outcome measures

Assessments are being conducted at three data collection time points (baseline, week 8 and week 20). The baseline assessment is conducted face-to-face at four sites located throughout metropolitan Adelaide, while the 8 week and 20 week assessments are conducted via the post. Assessments are delivered by non-blinded research personnel, however, there is minimal potential for bias given that the outcome measures are self-administered (i.e. surveys) by participants. Research personnel have been trained in the need to deliver the outcome measures in an impartial manner. Participants who complete all three assessments will receive $50 honoraria in recognition of the time and effort involved in undertaking the accelerometry and survey assessments.

At each assessment point, participants will complete the following measures:

1. Primary outcome: objective physical activity.

Objective physical activity is being assessed using Actigraph GT3X + accelerometers (ActiGraph, Pensacola, FL). The accelerometer is worn at the waist on an elasticized belt, on the right mid-axillary line. Participants are requested to use a wear-time log sheet to record time the device is put on and removed, as well as sleep and nap times. Since other studies have reported problems with meeting minimum-wear-time requirements with a waking hours protocol (i.e. daily instrument removal for sleep), participants are encouraged to wear the accelerometer 24 hours per day for 7 consecutive days. Accelerometers are initialized using ActiLife software [19], with an epoch length of 1 second and sampling rate of 80 Hz. Accelerometers are returned to the study site by reply-paid mail, at which time the research team verify the data for completeness using ActiLife software. Sixty minutes of consecutive zeros is being used to define invalid minutes (i.e. minutes in which the accelerometer was not worn). The minimal amount of accelerometer data that is considered acceptable is 4 days, including at least one weekend day, with at least 10 hours of valid wear time per day. If accelerometer data are incomplete, participants are asked to wear the accelerometer for an additional 7 days (to a maximum of 14 days) to ensure that the minimal data requirements are met. Accelerometers have been shown to accurately measure physical activity in children with physical disabilities (e.g. see [20,21]).

2. Secondary outcomes:

a. Exercise intention is being assessed using the LEAP II Exercise Intention Scale [22]. This scale was originally designed for 5th – 8th grade children. It examines intention to be physically active, and consists of four items rated on a 5-point scale, which are averaged to produce a single “Exercise Intention” score, ranging between 1 and 5, with a higher score indicating higher intention to be active. The factorial validity of this scale has been confirmed [22] and the scale has been used previously with children with cerebral palsy [11].

b. Physical self-worth is being assessed using the Physical Self-Worth Scale [23]. The scale contains 6-items, each scored from 1 to 4 (with 1 = 'very low’ self-worth and 4 = 'very high’ self-worth). Test-retest in 7th and 8th graders has been shown to be high (ICC = 0.86). The scale has been shown to correlate with physical fitness test scores to varying degrees (*r* = 0.23 - 0.57) [24] in adolescents without disability.

c. Quality of life (QOL) is being assessed using the KINDL [25], a generic QOL instrument designed for children aged 8–16. This 24 item scale records QOL in six dimensions, with the subscale scores combining to produce a total score. The KINDL has been shown to have moderate-to-high internal consistency (0.63 < α < 0.84), moderate convergent validity with other QOL instruments [25].

d. Fatigue is being assessed using the PedsQL Multidimensional Fatigue scale [26]. This generic instrument for children aged 5–18 contains 18 items assessing fatigue in three dimensions (general fatigue, sleep/rest and cognitive fatigue), with subscales tallied to produce an overall fatigue score. It has been demonstrated to have excellent reliability and construct validity in a variety of paediatric patient populations [26-28].

The surveys (outcome measures 2a-2d) are being delivered in pen and paper format.

### Randomisation

Random allocation to the intervention or control condition will take place after participants have completed all aspects of their baseline assessment.

Since age is recognised to impact children’s activity levels [29], and impairment level is associated with activity level in children with physical disabilities [9], a stratified randomised allocation procedure is being used to ensure an even balance of age and impairment levels between groups. Given the recognised effect of season on physical activity [30], blocking is being employed to ensure a close balance of participants in the intervention and control conditions throughout the study period, as recommended by CONSORT [31].

Randomisation is being achieved using four sets of opaque envelopes (Set 1: ages 8–12 years, Gillette level 7 or 8; Set 2: ages 13–17 years, Gillette level 7 or 8; Set 3: ages 8–12 years, Gillette level 9 or 10; Set 4: ages 13–17 years, Gillette level 9 or 10). Each set includes six envelopes, with three containing control allocation, and three containing intervention allocation. Randomisation is being undertaken by CM, who is provided with the participants’ ID number, age, and Gillette level, and who has no direct contact with participants.

### Description of the intervention

**Figure 1:**
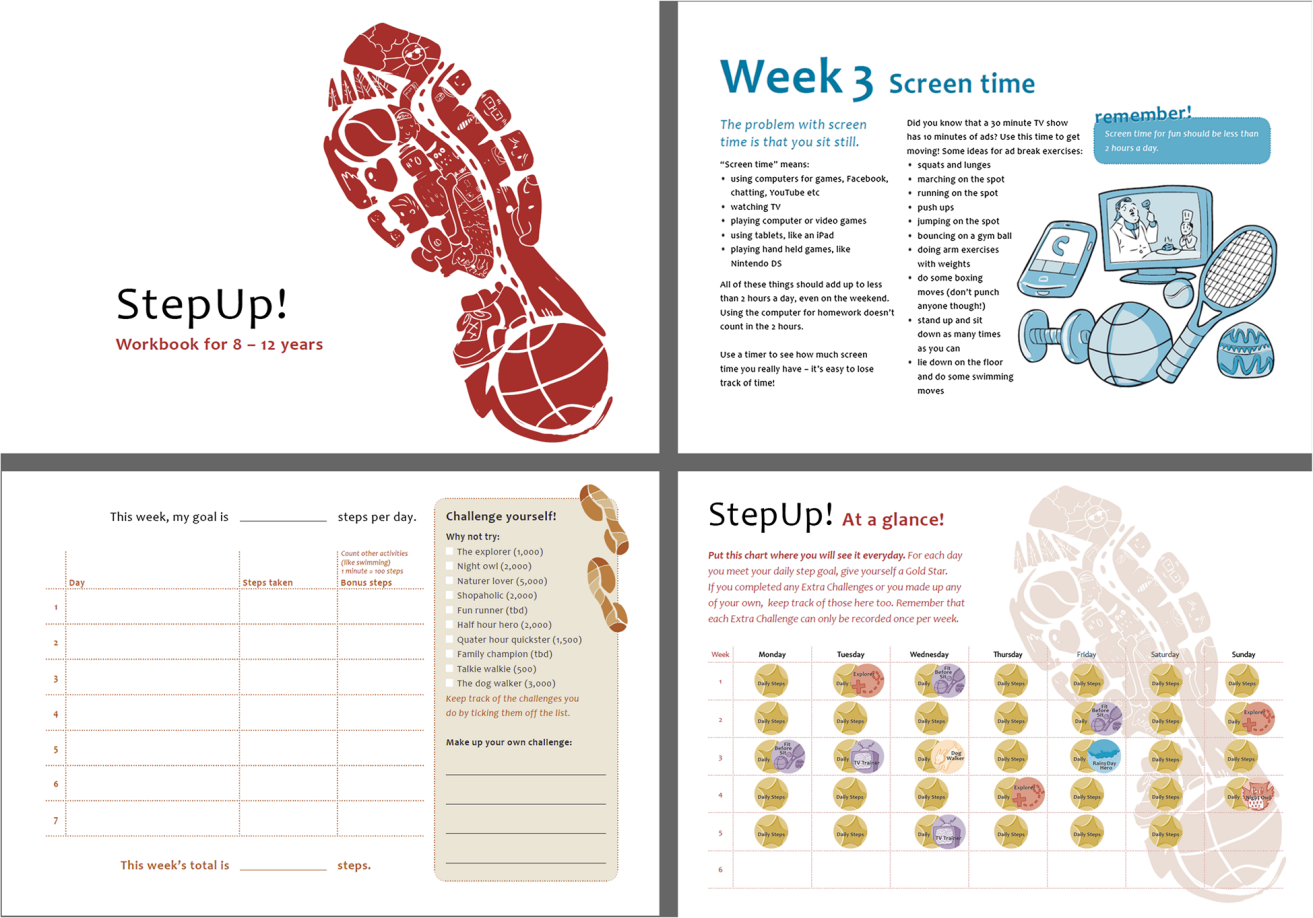
**Examples of the Step Up program materials.** Upper left panel: Step Up booklet cover; Upper right: example of weekly topic (“Screen time”); lower left: example of weekly log sheet for recording daily steps; lower right: wall sticker chart, including stickers for reaching daily step goal and stickers for extra challenges.

The “StepUp” intervention is a 6-week pedometer-based physical activity program for children with physical disabilities. It comprises two face to face visits and fortnightly phone follow ups with the physiotherapist, provision of a pedometer and StepUp handbook. There are two versions of the handbook - one for 8–12 year olds, and one for 13–17 year olds. The core content of the handbooks is the same, however the amount and complexity of the written content vary between the two age groups to accommodate the wide range of reading skills and age-appropriate content between the groups. Graphic design of the handbook has been undertaken by M Thiessen, who has expertise in the design and presentation of written information for children with reading difficulties, supplemented by illustrations from a professional illustrator. Further details of the intervention components and materials are provided in Table 1 and Figure 1. Week 1 of the StepUp program is being used to determine baseline activity levels (note this is distinct from the baseline outcome measurement period, which used an accelerometer to measure physical activity, and takes place prior to the participant being assigned to either the intervention or control group). Progressive daily targets are then negotiated by telephone between the physiotherapist and participant on a fortnightly basis.

**Table 1:** Description of the StepUp program

**Components**	**Description**
Contact with therapist	Week 1– face to face visit to introduce StepUp program
	Week 2 and week 4 – 5 minute phone call to negotiate weekly step count goals
	Week 6 - face to face visit to debrief re the StepUp program, and counsel re future plans for physical activity
Pedometer	MP-100 pedometer (Yamasa Corp; Chiba, Japan)
Step count targets	Step count targets for each week are negotiated with the therapist during the fortnightly phone call. Where the average daily step counts is < 6000 in the preceding week, the progressive goal aims to increase daily step counts by 10% compared with the previous week. Where the average daily step counts is > 6000 in the preceding week, the progressive goal aims to increase daily step counts by 5% compared with the previous week. Note that the 6,000 step cut off was based upon our previous research with children with cerebral palsy [9,11].
StepUp handbook educational info & weekly topic	Background info on using a pedometer, dealing with fatigue/pain/injuries, and how to contact the physiotherapist.
	Week 1 – “why be physically active”
	Week 2 – “how much is enough?”
	Week 3 – “Screen time”
	Week 4 – “Staying motivated”
	Week 5 – “Myth busters”
	Week 6 – “Step it up!”
StepUp challenges	The StepUp handbook contains a number of 'mini challenges’ which participants can complete, such as “Half Hour Hero” (for taking 2000 steps in 30 minutes), “Early Bird” (1000 steps before school), “Nature Lover” (5000 steps going for a nature walk), “Shopaholic” (2000 steps at a shopping centre) etc.
Tourist circuit	Steps are tallied at the end of each week, and young people can see how far they have walked (e.g. 55,000 steps = “Mad Marathon” (approx. 42 km); 450,000 steps = “Euro Tripper” (walking approx. 343 km, the distance from London to Paris).
Wall chart	Young people are encouraged to hang the wall chart in a prominent place (e.g. the fridge or their bedroom) and mark off, or use provided stickers, to chart when they have met their daily step goal, and when they have earned “badges” for the challenges and tourist circuit.
Rewards	Participants are encouraged to negotiate rewards with their parents for meeting their daily step count target. The parent booklet gives many ideas for rewards (including rewards which have no cost).
Parent booklet	Parents receive a booklet with information for supporting their child in the StepUp program including negotiating rewards, ideas for encouraging the child and the whole family to be active, and the physiotherapist’s contact details.

### Control condition

Participants randomised to the control condition are being told that their health will be monitored for 20 weeks, and are placed on a waiting list and provided with the full StepUp program at completion of the study.

### Program evaluation

Participants’ experiences and views regarding the StepUp program are being evaluated using a purpose-designed feedback survey (Additional file 1). At the end of the intervention, participants in the intervention group are invited to complete a 13-item feedback form, containing a combination of Likert-scale and open-ended items regarding what they thought of the various elements of the program, and what they liked, disliked and ideas for improvement. Participants’ daily step counts throughout the 6-week program are collected by the physiotherapist during the phone call and face-to-face appointments, and will be used to determine engagement and adherence with the program.

### Analysis

A priori power analyses indicate that a sample of 42 will be required to detect a medium size within-between group interaction (f = 0.20), while a sample of 74 will be required to detect a small-medium size within-between group interaction (f = 0.15), assuming three repeated measures, two groups, an alpha-level of 0.05 and power of 80%. The change in outcomes from baseline to 8 weeks and 20 weeks, within and between groups, will be analysed using random-effects mixed-modelling, on an intention-to-treat basis. The relationship between primary and secondary outcomes and demographic variables will be assessed and where relationships exist the demographic variables will be used as covariates.

Data from the feedback survey and engagement data will be analysed descriptively. In addition, sub-group analysis will be undertaken to determine whether the intervention effectiveness is related to engagement/adherence.

## Discussion

Physical activity is important for physical and psychological health, as well as for maintenance of physical function and independence in young people with physical disabilities. Despite this, there have been few rigorous studies aimed at intervening on physical activity in this population.

This project will evaluate the effectiveness of a novel pedometer-based physical activity self-management program for young people with physical disability.

Strengths and weaknesses of the StepUp study warrant discussion. The StepUp intervention has been carefully designed by a team of researchers and clinicians, which includes researchers with expertise in physical activity measurement, physical activity intervention, childhood physical disability, the development of written materials for children with learning difficulties, as well as highly experienced clinicians (physiotherapists and a psychologist). The program has been specifically designed to be low-cost and low-touch in nature, so that if it is found to be effective, it can be readily incorporated into ongoing clinical services. A strength of the study is that it involves a rigorous randomised controlled trial design, incorporating high quality outcome measures, including objective measurement of physical activity. The StepUp study is a single-site study. For budgetary and feasibility reasons, the study can only sample from a finite population (young people with physical disabilities meeting the eligibility criteria, including residing in Adelaide). We intend to invite the entire eligible population, and anticipate 40–70 participants will join the study, with a priori power analyses suggesting a sample of this magnitude should be sufficient to detect small to moderate effects, should they be present. For budgetary reasons, the follow up is only 20 weeks from baseline (12 weeks post end of intervention). Should the results of this study be affirmative, in the future it would be beneficial if the intervention could be evaluated in a larger sample, and over a longer-term period, in a multi-centre trial.

This study forms an early attempt at developing and evaluating a feasible physical activity intervention for children with physical disabilities, and findings will inform future efforts in this field, to assist young people with disabilities to achieve the benefits of physical activity.

## Competing interests

The authors declare that they have no competing interests that are directly related to the content of this manuscript.

## Authors’ contributions

CM conceived the study. All authors contributed to the protocol design.CM and MT and MT led the development of the StepUp materials, with input from all authors. MT oversaw participant recruitment and intervention delivery. CM led the drafting of this manuscript, with input from all authors. All authors have read and approved the final version of the manuscript.

## Pre-publication history

The pre-publication history for this paper can be accessed here:

http://www.biomedcentral.com/1471-2431/14/31/prepub

## Supplementary Material

Additional file 1 The Program Evaluation survey. Click here for file

## References

[B1] Trost S Discussion paper for the development of recommendations for children’s and youths’ participation in health promoting physical activity 2005 Canberra: Australian Department of Health and Ageing

[B2] Andersson C Mattsson E Adults with cerebral palsy: a survey describing problems, needs, and resources, with special emphasis on locomotion Dev Med Child Neurol 2001 43 76 82 10.1017/S0012162201 11221908

[B3] Jahnsen R Villien L Egeland T Stanghelle JK Holm I Locomotion skills in adults with cerebral palsy Clin Rehabil 2003 18 309 316 15137562 10.1191/0269215504cr735oa

[B4] Santiago MC Coyle CP Leisure-time physical activity and secondary conditions in women with physical disabilities Disabil Rehabil 2004 26 485 494 10.1080/09638280410001663139 15204471

[B5] Basaran S Guler-Uysal F Ergen N Seydaoglu G Bingol-Karakoç G Ufuk Altintas D Effects of physical exercise on quality of life, exercise capacity and pulmonary function in children with asthma J Rehabil Med 2006 38 130 135 16546771 10.1080/16501970500476142

[B6] Fredriksen PM Kahrs N Blaasvaer S Sigurdsen E Gundersen O Roeksund O Norgaand G Vik JT Soerbye O Ingjer E Thaulow E Effect of physical training in children and adolescents with congenital heart disease Cardiol Young 2000 10 107 114 10817293 10.1017/s1047951100006557

[B7] Australian Institute of Health and Welfare (AIHW) Children With Disabilities In Australia 2004 Canberra: Australian Institute of Health and Welfare

[B8] Law M King G King S Kertoy M Hurley P Rosenbaum P Young N Hanna S Patterns of participation in recreational and leisure activities among children with complex physical disabilities Dev Med Child Neurol 2006 48 337 342 10.1017/S0012162206000740 16608540

[B9] Maher CA Williams MT Olds T Lane AE Physical and sedentary activity in adolescents with cerebral palsy Dev Med Child Neurol 2007 49 450 457 10.1111/j.1469-8749.2007.00450.x 17518932

[B10] Maher C Kernot J Olds T Time use patterns in ambulatory adolescents with cerebral palsy Child Care Health Dev 2013 39 3 404 411 10.1111/j.1365-2214.2011.01352.x 22118515

[B11] Maher CA Williams MT Olds T Lane AE An internet-based physical activity intervention for adolescents with cerebral palsy: a randomized controlled trial Dev Med Child Neurol 2010 52 448 455 10.1111/j.1469-8749.2009.03609.x 20132138

[B12] Van Wely L Becher J Reinders-Messelink H Lindeman E Verschuren O Verheijden J Dallmeijer A LEARN 2 MOVE 7–12 years: a randomized controlled trial on the effects of a physical activity stimulation program in children with cerebral palsy BMC Pediatr 2010 10 77 10.1186/1471-2431-10-77 21044314 PMC2989952

[B13] Fowler EG Knutson LM DeMuth SK Sugi M Siebert K Simms V Azen SP Winstein CJ Pediatric endurance and limb strengthening for children with cerebral palsy (PEDALS)–a randomized controlled trial protocol for a stationary cycling intervention BMC Pediatr 2007 7 14 10.1186/1471-2431-7-14 17374171 PMC1838902

[B14] Slaman J Roebroeck M van Meeteren J van der Slot W Reinders-Messelink H Lindeman E Stam H van den Berg-Emons R LEARN 2 MOVE 16–24: effectiveness of an intervention to stimulate physical activity and improve physical fitness of adolescents and young adults with spastic cerebral palsy; a randomized controlled trial BMC Pediatr 2010 10 79 10.1186/1471-2431-10-79 21054829 PMC2992500

[B15] de Vries SI Bakker I Hopman-Rock M Hirasing RA van Mechelen W Clinimetric review of motion sensors in children and adolescents J Clin Epidemiol 2006 59 670 680 10.1016/j.jclinepi.2005.11.020 16765269

[B16] Lubans DR Morgan PJ Tudor-Locke C A systematic review of studies using pedometers to promote physical activity among youth Prev Med 2009 48 307 315 10.1016/j.ypmed.2009.02.014 19249328

[B17] Sawyer SM Aroni RA Self-management in adolescents with chronic illness. What does it mean and how can it be achieved? Med J Aust 2005 183 405 409 16225444 10.5694/j.1326-5377.2005.tb07103.x

[B18] Novacheck TF Stout JL Tervo R Reliability and validity of the gillette functional assessment questionnaire as an outcome measure in children with walking disabilities J Pediatr Orthop 2000 20 75 10641694

[B19] GT3X + Activity Monitor http://www.actigraphcorp.com/products/

[B20] Bjornson KF Belza B Kartin D Logsdon R Mc Laughlin JF Ambulatory physical activity performance in youth with cerebral palsy and youth who are developing typically Phys Ther 2007 87 248 257 10.2522/ptj.20060157 17244693 PMC1852474

[B21] Kuo YL Culhane KM Thomason P Tirosh O Baker R Measuring distance walked and step count in children with cerebral palsy: an evaluation of two portable activity monitors Gait Posture 2009 39 304 310 19019680 10.1016/j.gaitpost.2008.09.014

[B22] Motl R Dishman R Saunders R Dowda M Felton G Ward D Pate R Examining social-cognitive determinants of intention and physical activity among black and white adolescent girls using structural equation modeling Health Psychol 2002 21 459 467 12211513

[B23] Whitehead J Corbin C Effects of fitness test type, teacher, and gender on exercise intrinsic motivation and physical self-worth J Sch Health 1991 61 11 16 10.1111/j.1746-1561.1991.tb07850.x 2027288

[B24] Whitehead J A study of children’s physical self-perceptions using an adapted physical self-perception profile questionnaire Pediatr Exerc Sci 1995 7 132 132

[B25] Ernhart M Ellert U Kurth BM Ravens-Sieberer U Measuring adolescents’ HRQoL via self reports and parent proxy reports: an evaluation of the psychometric properties of both versions of the KINDL-R instrument Health Qual Life Outcomes 2009 26 77 10.1186/1477-7525-7-77 PMC2749015 19709410

[B26] Varni JW Burwinkle TM Katz ER Meeske K Dickinson P The PedsQL™ in pediatric cancer Cancer 2002 94 2090 2106 10.1002/cncr.10428 11932914

[B27] Varni JW Burwinkle TM Szer IS The PedsQL Multidimensional Fatigue Scale in pediatric rheumatology: reliability and validity J Rheumatol 2004 31 2494 2500 15570657

[B28] Varni JW Limbers CA Bryant WP Wilson DP The PedsQL™ Multidimensional Fatigue Scale in type 1 diabetes: feasibility, reliability, and validity Pediatr Diabetes 2009 10 321 328 10.1111/j.1399-5448.2008.00482.x 19067887

[B29] Nader PR Bradley RH Houts RM McRitchie SL O’Brien M Moderate-to-vigorous physical activity from ages 9 to 15 years JAMA 2008 300 295 305 10.1001/jama.300.3.295 18632544

[B30] Tucker P Gilliland J The effect of season and weather on physical activity: a systematic review Public Health 2007 121 909 922 10.1016/j.puhe.2007.04.009 17920646

[B31] Box 2 - Randomisation And Minimisation http://www.consort-statement.org/consort-statement/further-explanations/box2_randomisation_minimisation/

