# Supplementary material for: A pedometer based physical activity self-management program for children and adolescents with physical disability – design and methods of the StepUp study
Source: BMC Pediatr. 2014 Feb 3;14:31. doi: 10.1186/1471-2431-14-31 (PMC3930302; doi:10.1186/1471-2431-14-31)
Supplement: Additional file 1 — The Program Evaluation survey. [file 1471-2431-14-31-S1.docx]

Additional file 1 – the Program Evaluation survey

|  | Disagree a lot | Disagree a little | | Neither disagree nor agree | Agree a little | | Agree a lot |
| --- | --- | --- | --- | --- | --- | --- | --- |
| 1. I think the StepUp program was easy to use. |  |  | |  |  | |  |
| 2. I liked the appearance of the StepUp program book, wall chart & stickers. |  |  | |  |  | |  |
| 3. I think the information each week was interesting and useful. |  |  | |  |  | |  |
| 4. I remembered to write down my steps each day. |  |  | |  |  | |  |
| 5. The pedometer worked well for me (was accurate & easy to use). |  |  | |  |  | |  |
| 6. It was helpful having regular contact with the physio (by email / phone). |  |  | |  |  | |  |
| 7. I thought the challenges were interesting. |  |  | |  |  | |  |
| 8. Doing the StepUp program helped me do more physical activity. |  |  | |  |  | |  |
| 9. I plan to continue using the pedometer after the StepUp program finishes. |  |  | |  |  | |  |
| 10. I thought 6 weeks for the StepUp program was: | Too short | | About right | | | Too long | |
| 11. I thought the step count targets each week were: | Very easy | A bit easy | | About right | A bit hard | | Very hard |
| 12. What did you like best about the StepUp program? |  | | | | | | |
| 13. What did you like least about the StepUp program? |  | | | | | | |
| 14. What would you change about the StepUp program if you could? |  | | | | | | |
